# Stress-Activated Protein Kinases in Intervertebral Disc Degeneration: Unraveling the Impact of JNK and p38 MAPK

**DOI:** 10.3390/biom14040393

**Published:** 2024-03-25

**Authors:** Lei Li, Guangzhi Zhang, Zhili Yang, Xuewen Kang

**Affiliations:** 1 Department of Orthopedics, Lanzhou University Second Hospital, Lanzhou 730030, China; lil21@lzu.edu.cn (L.L.); zhanggzh18@lzu.edu.cn (G.Z.); 220220904381@lzu.edu.cn (Z.Y.); 2 The Second Clinical Medical College, Lanzhou University, Lanzhou 730030, China; 3 Key Laboratory of Orthopedics Disease of Gansu Province, Lanzhou University Second Hospital, Lanzhou 730030, China; 4 The International Cooperation Base of Gansu Province for the Pain Research in Spinal Disorders, Lanzhou 730030, China

**Keywords:** intervertebral disc degeneration, JNK, p38 MAPK, SAPKs, stress signaling

## Abstract

Intervertebral disc degeneration (IDD) is a major cause of lower back pain. The pathophysiological development of IDD is closely related to the stimulation of various stressors, including proinflammatory cytokines, abnormal mechanical stress, oxidative stress, metabolic abnormalities, and DNA damage, among others. These factors prevent normal intervertebral disc (IVD) development, reduce the number of IVD cells, and induce senescence and apoptosis. Stress-activated protein kinases (SAPKs), particularly, c-Jun N-terminal kinase (JNK) and p38 mitogen-activated protein kinase (p38 MAPK), control cell signaling in response to cellular stress. Previous studies have shown that these proteins are highly expressed in degenerated IVD tissues and are involved in complex biological signal-regulated processes. Therefore, we summarize the research reports on IDD related to JNK and p38 MAPK. Their structure, function, and signal regulation mechanisms are comprehensively and systematically described and potential therapeutic targets are proposed. This work could provide a reference for future research and help improve molecular therapeutic strategies for IDD.

## 1. Introduction

Musculoskeletal disorders are among the leading causes of disabilities worldwide. According to a recent statistical analysis, approximately 1.71 billion people worldwide have been diagnosed with musculoskeletal diseases, with the proportion of patients with low back pain (LBP) being as high as 33% [1]. With an increasingly aging population, LBP places a substantial economic burden on global health systems [2]. Intervertebral disc degeneration (IDD) is a significant factor associated with the onset of LBP [3]. The early treatment of IDD primarily depends on physical interventions and medications to achieve conservative efficacy. However, the later stages of the disease, marked by annulus fibrosus (AF) avulsion, cartilaginous endplate (CEP) collapse, and severe nucleus pulposus (NP) prolapse within the intervertebral disc (IVD), necessitate surgical intervention for pain relief and partial motor function restoration [4]. However, the current therapies for IDD mainly alleviate symptoms and fail to reverse the underlying pathological process. Therefore, studying the molecular mechanisms underlying the pathophysiology of IDD may provide a new strategy for treating IDD.

The pathogenesis of IDD is complex and not fully understood but is closely associated with the stimulation of various stressors, such as proinflammatory cytokines [5], abnormal mechanical stress [6], oxidative stress [7], metabolic abnormalities [8], and DNA damage [9], among others. These factors affect and prevent the normal development of the IVD, reduce the number of IVD cells, induce senescence, apoptosis, and extracellular matrix degradation, and accelerate degeneration.

Stress-activated protein kinases (SAPKs) are cellular signaling control elements that respond to cellular stress and are dominated by c-Jun N-terminal kinase (JNK) and p38 mitogen-activated protein kinase (p38 MAPK) [10]. JNK and p38 MAPK are found in various human tissues, including the lungs, liver, immune organs, fat, skeletal muscles, and the central nervous system. The activation of JNK and p38 MAPK signaling regulates various cellular activities, including cell proliferation, senescence, autophagy, migration, death, and osmotic pressure [11,12,13,14]. Thus, JNK and p38 MAPK are involved in the development of multiple diseases, such as cancer, Parkinson’s disease, Alzheimer’s disease, amyotrophic lateral sclerosis, spinal cord injury, etc. [15].

Previous studies have shown that JNK and p38 MAPK are highly expressed in degenerated IVD tissues and involved in complex biological signal-regulated processes [16]. As a signal transduction pathway in IVD cells, JNK and p38 MAPK play important roles in regulating extracellular matrix (ECM) metabolism, inflammation, senescence, apoptosis, oxidative stress damage, ferroptosis, and autophagy by receiving signals from intracellular and extracellular stress stimuli. For instance, studies have demonstrated that JNK and p38 MAPK can significantly promote ECM catabolism and inhibit anabolism in degenerated NP cells, thereby disrupting the balance of the ECM environment [17,18]. Similarly, the activation of JNK and p38 MAPK is required for the release of pro-inflammatory cytokines in degenerated tissues [19]. As IDD progresses, various stress stimuli can enhance the activity of JNK and p38 MAPK, as well as increase the number of senescent cells [20,21]. In degenerate IVD cells, JNK and p38 MAPK promote apoptosis and initiate the programmed death process [22,23]. JNK and p38 MAPK can also increase the content of intracellular reactive oxygen species (ROS), leading to oxidative stress damage, and affect the excessive accumulation of iron in cells, leading to the occurrence of ferroptosis [24,25]. Additionally, inhibition of JNK and p38 MAPK significantly increases the level of intracellular autophagy in NP cells, thus delaying apoptosis and ECM degradation [26,27]. However, the specific signal transduction mechanism of JNK and p38 MAPK in IDD is not clear and needs further exploration.

Therefore, we summarize the research reports on IDD related to JNK and p38 MAPK. The structure, function, and signal regulation mechanisms of these two SAPKs are described comprehensively and systematically and their potential therapeutic targets are proposed. A better understanding of the precise mechanisms underlying IDD may guide the development of molecular therapeutic strategies and provide a reference for future studies.

## 2. The Basic Structure and Function of the IVD

**Figure 1:**
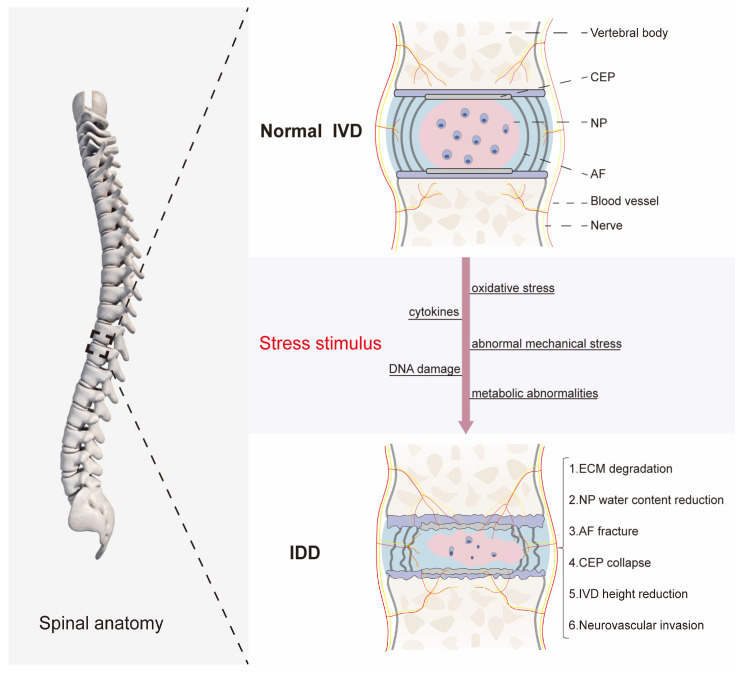
The normal structure of the intervertebral disc and the pathological process of degeneration. The normal intervertebral disc (IVD) consists of the nucleus pulposus (NP), annulus fibrosus (AF), and cartilaginous endplate (CEP). Under the stimulation of various stressors, the microenvironment in IVD changed, along with the degradation of the extracellular matrix (ECM), the loss of water in NP, and the decrease in cell number. AF was torn and accompanied by NP protrusion. CEP collapse and IVD height could not be maintained. Nerves and blood vessels intrude into the IVD. These factors accelerate the development of intervertebral disc degeneration (IDD).

The IVD, as part of the spine structure, plays a significant role as a shock absorber for the body’s natural axial load, absorbing biological forces and maintaining the body’s three-dimensional range of motion [28]. The IVD is the largest avascular tissue in the human body and consists of three parts: the gelatinous NP in the central region, surrounding lamellar AF, and CEP connected to the upper and lower vertebrae (Figure 1) [29]. NP mainly comprises proteoglycans and type II collagen. Proteoglycans maintain hydrostatic pressure by acting as hydrogels and by adsorbing water molecules [30]. Type II collagen is the main component of the NP collagen network and supports the entire NP. The structure of NP enables it to resist stress and maintain elasticity, compressibility, and flexibility. AF mainly comprises a highly structured network of collagen fibers dominated by type I collagen. AF is characterized by its tensile properties, which stabilize the location of NP tissue and play a specific role in nutrient transport [31]. The CEP is a layer of hyaline cartilage less than 1 mm thick and difficult to detach [32]. The majority of studies on IDD primarily focus on NP and AF. However, CEP is critical for maintaining nutrient transport in normal IVD, with most small-molecule nutrients being delivered specifically via CEP [33]. A comprehensive analysis of the structural components of IVD reveals that an intact IVD tissue is viscoelastic and capable of resisting compression-tensile stress and shear forces, which are structural features necessary for maintaining regular human activity [28,29,34].

## 3. The Pathological Mechanism of IDD

The manifestation of IDD is a long and complex process that is typically associated with a healthy IVD undergoing degenerative alterations and involves multiple factors. This complex pathological mechanism is intricately related to morphological and intrinsic physiological changes in the three basic structures of the IVD, including AF, NP, and CEP. There is still an ongoing controversy surrounding whether the AF, NP, or CEP serves as the initial site of the lesion. However, since NP is located in the central region of the IVD in a closed environment that is ischemic and hypoxic, it is generally accepted that alterations in the NP microenvironment may potentially influence the degenerative process at the initial stage of IDD development [35]. This microenvironmental alteration is primarily manifested through substantial alterations in the ECM, which have significant implications for the normal development, degeneration, and regeneration of IVD [36]. Under normal circumstances, the ECM in IVD cells is in a state of dynamic equilibrium. When stimulated by various stressors (proinflammatory cytokines, abnormal mechanical stress, oxidative stress, and DNA damage, among others), the imbalance between the anabolism and catabolism of the ECM causes microenvironmental damage, leading to the loss of tissue repair capacity of IVD [37,38]. During the development of IDD, the synthesis of proteoglycans and type II collagen in NP tissues is inhibited, whereas the expression of matrix degradation molecules, including matrix metalloproteinases (MMPs), as well as a disintegrin and metalloproteinase with thrombospondin motifs (ADAMTS), is enhanced. A reduction in proteoglycans within the NP results in water loss, eventually leading to the failure to maintain its normal morphology and biomechanical properties. These factors constitute critical links in the metabolic derangement of the ECM [39]. Persistent degradation of type II collagen and an increase in type I collagen lead to increased ECM stiffness within the nucleus pulposus cells (NPCs) and loss of the boundary between the NP and AF [40]. The arrangement of the collagen fiber network and distribution of glycosaminoglycan (GAG) in the AF contribute to the generation of residual stresses and strains, which are compressed in the inner ring and stretched in the outer ring. Loss of GAG in the inner ring of the IVD tissues with mild to moderate degeneration reduces the expansion volume and circumferential stress by more than 50% [41]. This degeneration of the AF results in an alteration in the radial force from compression to tension, which may be one of the crucial reasons for the protrusion of NP due to the formation of inner tears in AF during the development of IDD. Calcification and sclerosis of the CEP are other important factors that substantially contribute to the acceleration of IDD. The nutrition of NP depends on the transport mediated by CEP. CEP calcification and hardening limit substance exchange between NP and CEP due to the reduced permeability and porosity of the CEP matrix [42]. Inadequate nutrient supply and the continuous buildup of metabolite waste lead to the death of NPCs unable to meet their normal growth requirements. Concurrently, the loss of the surrounding ECM accelerates the overall degradation process (Figure 1).

In summary, the pathological changes in IDD are inseparable from the stimulation of various stressors. JNK and p38 MAPK are the two major SAPKs that respond to and transmit stress signals from the extracellular domain, thereby participating in disease development. Therefore, it is vital to investigate the role of JNK and p38 MAPK in the activation of IVD cells under stress stimuli and the effect of signal transduction after activation on the disease.

## 4. Activation and Signaling of JNK and p38 MAPK

**Figure 2:**
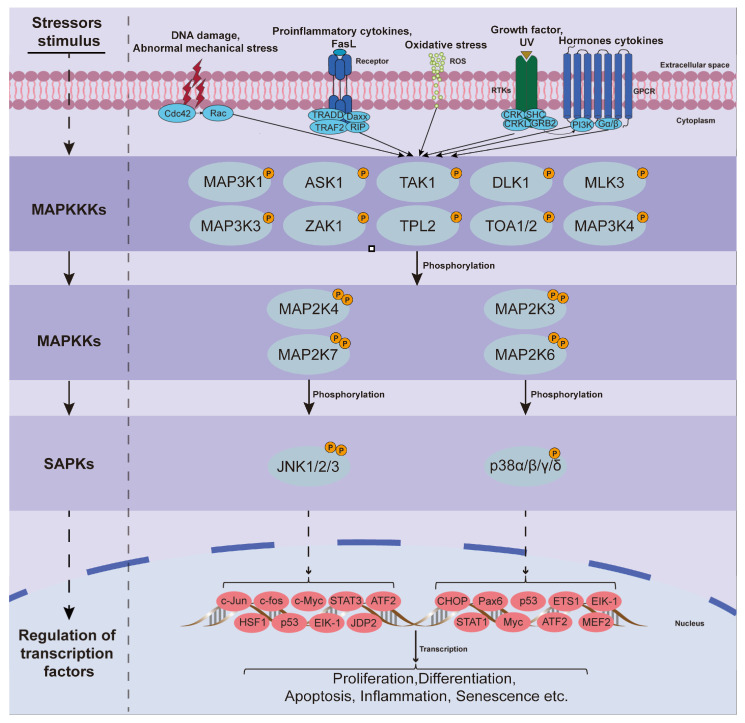
Activation and transmission of JNK and p38 MAPK signals. Under a variety of stress stimuli (such as oxidative stress, DNA damage, aberrant mechanical stress, proinflammatory cytokines, etc.), extracellular-derived stress signals are introduced into the cell via the corresponding membrane receptors on the cell membrane and are amplified by the MAPKKKs-MAPKKs-MAPKs cascade effect. Bisphosphonated-activated JNK and p38 MAPK enter the nucleus and act on related transcription factors to regulate pathophysiological processes such as cell proliferation, differentiation, apoptosis, inflammatory response, and senescence. ASK1, Signal-regulating kinase 1; CHOP, C/EBP homologous protein; DLK1, Dual-leucine-zipper-bearing kinase 1; TAK1, Transforming growth factor β-activated kinase 1; TOA1/2, Thousand and one amino acids 1 and 2; TPL2, Tumor progression locus 2; MLK3, Mixed-lineage kinase 3; ZAK1, Leucine zipper and sterile-α motif kinase 1; STAT3, Signal transducer and activator of transcription 3; ATF2, Activating transcription factor 2; Eik-1, Eukaryotic initiation factor-1; HSF1, Heat shock factor 1; JDP2, Jun dimerization protein 2; MEF2, Myocyte enhancer factor 2.

The mitogen-activated protein kinase (MAPK) family members in mammalian cells include extracellular regulated protein kinase (ERK), JNK, and p38 MAPK. ERK is primarily activated by extracellular mitogens and growth factors, whereas JNK and p38 MAPK are activated by stress stimuli and are classified as SAPKs. The MAPK signaling cascade is generally activated by the phosphorylation of three kinases in sequence to ensure signal amplification and fidelity, including MAPK kinase kinases (MAPKKKs), MAPK kinases (MAPKKs), and MAPKs (Figure 2) [13].

### 4.1. JNK

JNK, a novel serine/threonine protein kinase, with molecular weights ranging between 46 and 55 kD, was discovered by Kyriakis et al. in 1990 [43]. This protein kinase primarily comprises 11 subunits, which form a conserved three-dimensional folding structure of the protein and exhibit the following specific structural features: (1) the carboxy-terminal protrusions, which are dominated by the α-helix structure and mainly function as substrates for recognition and anchoring the phosphate group on Mg2^+^-ATP and (2) the amino-terminal protrusions, which are dominated by the reversed β-sheet structure and function as sites for localization and binding of ATP [44,45]. The JNK family includes three members encoded by different genes, including JNK1, JNK2, and JNK3 [46]. Although their amino acid sequence identity is >85%, their distribution in the human body varies. JNK1 and JNK2 are widely expressed in human tissues, whereas JNK3 is only expressed in the brain, testes, and heart [47]. JNK are generally located in the cytoplasm, whereas only a few are located in the nucleus. However, when stress signals stimulate cells, JNK is rapidly transmitted to the nucleus after activation and participates in the corresponding gene expression changes.

Although genes encoding JNK can give rise to 10 different JNK subtypes via alternative splicing, all of these subtypes contain a threonine-proline-tyrosine (Thr-Pro-Tyr) motif [48]. This motif plays a central role in JNK activation. JNK activation is accomplished by dual phosphorylation of Thr and Tyr in this specific motif structure, which is mediated by a tertiary phosphorylation cascade response [49]. First of all, stress stimulus from outside the cell, for instance, oxidative stress injury, ionizing radiation, heat shock, osmotic pressure, cytokines (such as tumor necrosis factor-alpha (TNF-α), cluster of differentiation (CD28) and interleukin-1 beta (IL-1β)), and biological factors (such as DNA and epidermal growth factor (EGF)), and certain G protein-coupled receptors (GPCRs) activate the phosphorylation of MAPKKKs (specifically MAP3K1). Secondly, after the activation of tertiary kinase MAPKKKs, the secondary kinase MAPKKs are further activated by phosphorylation. Finally, MAPKKs activate JNK through bisphosphorylation of Thr and Tyr, which is hypothesized to be explicitly mediated by MAP2K4 and MAP2K7 [11,50]. Activated JNK enters the nucleus to regulate various transcription factors, including c-Jun, c-fos, p53, signal transducer and activator of transcription (STAT)3, activating transcription factor (ATF)2, SMAD4, eukaryotic initiation factor (Eik)-1, Jun dimerization protein (JDP2), heat shock factor (HSF)1, c-Myc, and others. Similarly, multiple framework proteins and protein kinases associated with signaling and apoptosis-related proteins are directly regulated in the cytosol [51]. After activation, these substrates participate in the pathophysiological processes of cells, tissues, and organs by activating different signaling pathways and interacting with each other, causing cell proliferation, tissue differentiation, apoptosis, inflammatory responses, and senescence, thereby affecting the development of diseases (Figure 2).

### 4.2. p38 MAPK

p38 MAPK was first discovered by Brewster et al. in 1993 while studying the effects of osmotic changes on yeast. Subsequently, Han et al. determined that the alteration of intracellular osmolarity in mammalian cells, induced by lipopolysaccharide (LPS) exposure, triggers a response from p38 MAPK. Notably, p38 MAPK shares similarities with JNK and belongs to the same genus as SAPKs [52,53]. The p38 MAPK family comprises four isoforms encoded by different genes located in tandem on two chromosomes, namely p38 alpha (p38α, SAPK2A), p38 beta (p38β, SAPK2B), p38 gamma (p38γ, SAPK3), and p38 delta (p38δ, SAPK4) [54]. Because of their highly similar biomolecular structure, the p38 MAPK family is grouped into two subgroups, in which p38α and p38β belong to one subgroup as they share 75% sequence identity. Similarly, p38γ and p38δ belong to another subgroup because of 75% amino acid sequence identity [55]. The isoform, p38α, is the most well-known isomer of p38 MAPK and is widely distributed in the human body. The distribution of the other three subtypes is specific, with p38β mainly distributed in the brain, thymus, and spleen; p38γ exhibiting abundance in skeletal muscle; and p38δ primarily expressed in the pancreas, lungs, small intestines, kidneys, and testes [54,56]. Interestingly, p38 MAPK isoforms are also differentially expressed in the degenerated IVD tissues. Yang et al. [57] observed that the protein expression levels of p38 MAPK in NPCs were significantly higher in comparison to AF in human-derived IDD tissues. Subsequently, they compared the protein expression differences of the four subtypes of p38 MAPK in degenerated versus normal NP tissues and found that both p38α and p38β were highly expressed in the degenerated tissues with no significant difference between them, whereas p38δ was expressed only in half of the degenerated specimens. However, the expression of p38γ was either significantly low or absent in all the degenerated tissue samples. The differential expression of specific subtypes may offer valuable insights, enabling precise targeting of p38 MAPK-targeted molecules for the treatment of IDD.

In most cases, the activation conditions of the p38 MAPK pathway are similar to those of the JNK pathway and stress stimuli that activate JNK can also activate p38 MAPK. Similar to the JNK activation process, p38 MAPK exhibits a unique motif in its three-dimensional structure, referred to as threonine-glycine-tyrosine (Thr-Gly-Tyr) [58] and is located on the activation ring of the kinase subdomain VIII. The activation of p38 MAPK occurs through the dual phosphorylation of Thr and Tyr on this motif. Bisphosphorylation at these two specific sites induces a conformational change in p38 MAPK by stabilizing the activation loop in a more open configuration and promoting rotation between the two major leaflets [59,60,61]. This enables substrate recognition and increased kinase activity. The MAPKKs that phosphorylate p38 MAPK are MAP2K3, MAP2K4, and MAP2K6. However, only MAP2K3 and MAP2K6 specifically activate p38 MAPK [62]. Activation of MAP2K3 and MAP2K6 is mediated by MAP3Ks-mediated phosphorylation of serine (Ser) and Thr residues. Notable MAP3Ks involved in this process include apoptosis signal-regulating kinase 1 (ASK1), dual-leucine-zipper-bearing kinase 1 (DLK1), transforming growth factor β-activated kinase 1 (TAK1), thousand and one amino acids 1 and 2 (TOA1/2), tumor progression locus 2 (TPL2), mixed-lineage kinase 3 (MLK3), leucine zipper and sterile-α motif kinase 1(ZAK1), MAP3K3, and MAP3K4 [63]. Thus, the diversity of MAP3Ks capacitates cells to respond to various stimuli. The p38 MAPK is activated by a wide range of stimuli, which potentially involve various signaling elements and substrates and lead to distinct cellular effects (Figure 2).

## 5. JNK and p38 MAPK Signaling Pathways Are Involved in the Process of IDD

**Figure 3:**
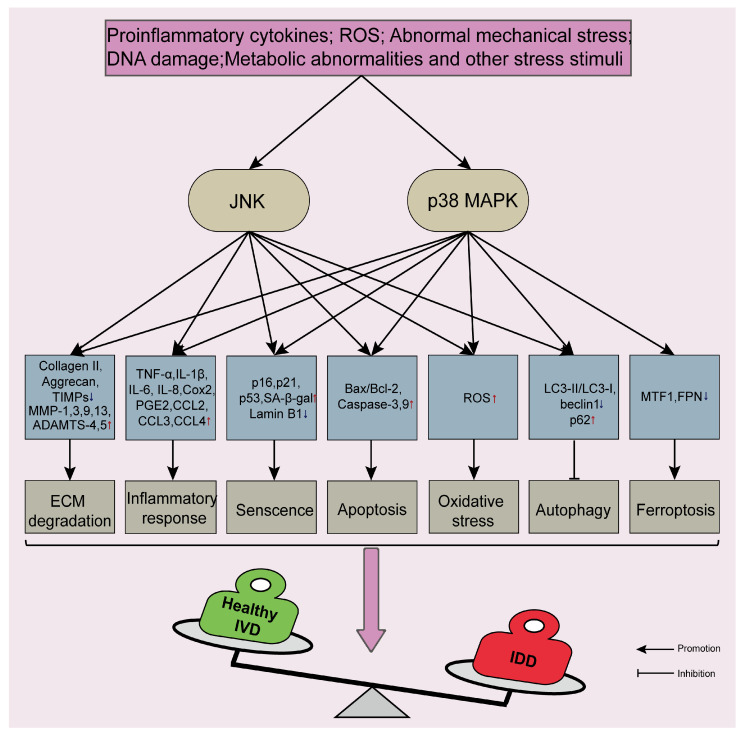
Potential regulatory mechanisms of JNK and p38 MAPK signaling pathways in intervertebral disc degeneration. When multiple stressors stimulate intervertebral disc (IVD) cells, JNK and p38 MAPK signaling pathways are significantly activated and involved in regulating extracellular matrix (ECM) metabolic balance, inflammatory response, senescence, apoptosis, oxidative stress injury, ferroptosis, and autophagy in IVD cells. Ultimately, healthy IVD will suffer from degeneration irreversibly under these influences. TIMPs, Tissue inhibitor of metalloproteinase; MMPs, Matrix metalloproteinase proteins; ADAMTS, A disintegrin and metalloproteinase with thrombospondin motifs; TNF-α, Tumor necrosis factor-alpha; IL-1β, Interleukin-1 beta; CCL3, Chemokine (C-C motif) ligand 3; PGE2, Prostaglandin E2; SA-β-gal, Senescence-associated β-galactosidase; ROS, Reactive oxygen species; LC3, Microtubule-associated protein 1 light chain 3; MTF1, Metal-regulated transcription factor 1; FPN, Ferroportin; IDD, Intervertebral disc degeneration. The red arrows represent up-regulation and the blue arrows represent down-regulation.

The pathophysiological process of IDD is regulated by multiple intracellular signaling pathways, including the common nuclear factor kappa B (NF-κB), mammalian target of rapamycin (mTOR), phosphoinositide 3-kinase/protein kinase B (PI3K/AKT), AMP-activated protein kinase (AMPK), and mitogen-activated protein kinase (MAPK) signaling pathways [40,64,65,66,67]. The activation of these pathways is selective to a certain extent and the regulatory relationships between these pathways are complex. The JNK and p38 MAPK signaling pathways are branches of the MAPK signaling pathway, which regulate various cellular activities by responding to stress stimuli. Studies have shown that the expression levels of JNK and p38 MAPK gradually increase with the exacerbation of IDD [16,57,68]. When multiple stressors stimulate IVD cells, the JNK and p38 MAPK signaling pathways are significantly activated. These pathways play vital roles in regulating the metabolic balance of ECM, inflammatory response, senescence, apoptosis, oxidative stress injury, ferroptosis, and autophagy in IVD cells (Figure 3).

### 5.1. JNK and p38 MAPK Signaling Pathways Disrupt ECM Metabolic Balance and Reduce ECM Content

Disruption of ECM metabolism is regarded as an essential pathological process of IDD, influenced by multiple factors that induce an abnormal upregulation of MMPs (MMP-1, -2, -3, -7, -8, -9, -10, and-13) and ADAMTS (ADAMTS-1, -4, and-5). This causes excessive degradation of ECM components and ultimately results in the loss of essential biological properties in the IVD [69]. Activation of the JNK and p38 MAPK signaling pathways in degenerated IVD tissues leads to the upregulation of MMPs and ADAMTS, accompanied by a downregulation of aggrecan and type II collagen expression. This alteration is particularly pronounced in the NP tissues.

TNF-α and IL-1β are the most widely studied proinflammatory cytokines and can reduce ECM content by activating JNK and p38 MAPK signaling pathways [17,18]. Daniels et al. [18] identified three signaling pathways activated by IL-1β in degenerated IVD tissues, including JNK, p38 MAPK, and NF-κB. Inhibition of their activity leads to a significant reduction in the effect of IL-1β on ECM degradation. Resistin is a polypeptide hormone secreted primarily by adipocytes that promotes inflammation and catabolism during cartilage metabolism. Liu et al. [70] revealed that resistin could increase ADAMTS-5 expression in rat NPCs in a time- and dose-dependent manner. The p38 MAPK signaling pathway is activated upon exposure to resistin, whereas treatment with a p38 MAPK inhibitor reduces the upregulation of ADAMTS-5 by resistin. Similarly, leptin is a hormone secreted by adipocytes that regulates biological behavior and metabolism. Leptin significantly induces p38 MAPK phosphorylation in human NPCs; in turn, the activated p38 MAPK accelerates aggrecan degradation in NPCs by upregulating the expression of ADAMTS-4 and ADAMTS-5 [71]. Miao et al. [72] conducted similar animal studies and confirmed that leptin can elevate the expression levels of the catabolic markers MMP-1, MMP-13, ADAMTS-4, and ADAMTS-5. Simultaneously, the expression levels of type II collagen were found to decrease and this response was closely associated with the activation of the JNK and p38 MAPK signaling pathways. In addition to NPCs, leptin and its receptors are also expressed in AF cells. Ding et al. found that leptin increases the protein expression levels of type X collagen and MMP-13 in rat AF cells via the p38 MAPK pathway; however, the activity of JNK was not regulated during this process [73].

Rab7 is a small G protein in the superfamily of RAS proteins that assists in vesicle transport and participates in signal transduction. Chen et al. [74] demonstrated that Rab7 is under expressed in human degenerated NP tissues and that overexpression of Rab7 can inhibit the p38 MAPK signaling pathway. Restriction of p38 MAPK phosphorylation reduces MMP production and ECM degradation in NPCs, thus delaying the degenerative process of IVD. Lactoferrin is an iron-binding protein with a molecular weight of approximately 80kD. It exhibits multiple biological effects, including antibacterial, antiviral, and antitumor effects, and regulates cell growth and metabolism. A previous study indicated that lactoferrin in bovine-derived NPCs increased the synthesis of proteoglycan and tissue inhibitor of metalloproteinase (TIMPs) and downregulated the expression of catabolic markers through a dose-dependent inhibition of the p38 MAPK signaling cascade pathway [75]. Syndecan-4 is a transmembrane proteoglycan that belongs to the poly ligand proteoglycan family of heparan sulfate proteoglycans and plays a vital role in the development of IDD. Ge et al. [76] observed that syndecan-4 effectively activates JNK and its downstream p53 pathway, which is accompanied by decreased protein expression levels of type II collagen and aggrecan and increased expression levels of collagen X, which exacerbates the IDD process. Notably, this result was reversed after treating the cells with the JNK inhibitor, SP600125. High-temperature serine protease A1(HTRA1) is a secretory serine endopeptidase that plays an essential physiological role in extracellular matrix protein denaturation and is associated with the development of diseases such as arthritis, age-related macular degeneration, and Alzheimer’s disease. Li et al. [77] examined the changes in ADAMTS by adding exogenous HTRA1 to human nucleus pulposus cells (HNPCs) and found that HTRA1 increased ADAMTS-5 expression in a dose- and time-dependent manner, whereas no significant increase was observed in ADAMTS-4 expression. However, exogenous HtrA1-induced ADAMTS-5 was significantly reduced upon treatment with ERK/NF-ΚB/JNK signaling pathway inhibitors. In addition, JNK phosphorylation was higher in denatured CEP cells than in normal cells, whereas type II collagen and aggrecan phosphorylation were lower in the ECM components. However, when JNK phosphorylation is inhibited, its expression levels increase significantly [68]. In conclusion, JNK and p38 MAPK signaling pathways play a significant role in regulating ECM homeostasis in IDD. Further research is crucial to gain deeper insights into their specific regulatory mechanisms.

### 5.2. JNK and p38 MAPK Signaling Pathways Regulate the Inflammatory Response

The inflammatory response in IDD is considered a critical factor in accelerating degeneration, which is governed by hierarchical imbalanced proinflammatory cytokines networks [78]. During degeneration, multiple proinflammatory cytokines, such as TNF-α, IL-1β, IL-6, IL-8, and IL-17, and chemokines, such as chemokine (C-C motif) ligand (CCL)2, CCL3, and chemokine (C-X-C motif) ligand (CXCL10), are extensively activated, leading to the so-called low-grade chronic inflammatory response with a crucial role in LBP development [5,79]. These proinflammatory cytokines promote a catabolic response, leading to ECM loss, while simultaneously promoting macrophage (MΦ) migration and infiltration into the IVD, which amplifies the inflammatory response [80]. Activation of JNK and p38 MAPK is required for MΦ-induced proinflammatory cytokine release in IDD. Park et al. [19] co-cultured macrophage-like THP-1 cells with human-derived AF and NP cells and found higher secretion of IL-6 and IL-8 in co-cultured cells than in cells cultured alone. Interestingly, TNF-α and IL-6 expression levels were significantly lower in co-cultured NPCs than in co-cultured AF cells, which may be related to the preferential invasion of AF tissue by vessels towards NP during IDD. The expression levels of TNF-α, IL-6, and IL-8 were significantly reduced in AF-MΦ and NP-MΦ co-cultured cell systems when JNK and p38 MAPK inhibitors were used. In addition, the JNK inhibitor also significantly down-regulated the expression of IL-1β in the NP-MΦ co-culture system. Similarly, Ni et al. [81] investigated how pro-inflammatory M1-type cells in MΦ participate in IDD. A degeneration model was established by treating rat NPCs with conditioned media from M1-polarized RAW264.7 cells (MΦCM), which showed that MΦCM disrupted ECM metabolic balance and upregulated the transcription of inflammation-related genes (IL-1β, IL-6, CCL2, and CCL3). MΦCM significantly increased JNK phosphorylation but the expression of catabolism and inflammatory genes was restricted when the JNK pathway was inhibited. The migration of MΦ is inevitably associated with the induction of chemokines. Resistin has been shown to upregulate CCL4 and promote the binding of CCL4 to its receptor, CCR1, thereby accelerating MΦ migration. Further studies demonstrated that the increased expression of CCL4 was partly due to increased transcription of CCL4 by resistin through activation of p38 MAPK in the nucleus; contrastingly, the inhibition of p38 MAPK significantly reduced the expression of CCL4 and inhibited the inflammatory response induced by MΦ infiltration [82].

In addition, SAPKs regulate the upstream transcription factors of pro-inflammatory cytokines. CCAAT/enhancer binding protein β (C/EBP β) is one of the transcription factors upstream of TNF-α, which regulates its expression. Studies have shown that p38 MAPK inhibitors in rat NPCs block the expression of TNF-α transcription factor C/EBP β, inhibit TNF-α synthesis, and alleviate inflammatory responses within NPCs [83]. However, JNK inhibitors are ineffective and this differential effect may be related to JNK substrate selectivity. Cytokines can aggravate or alleviate inflammatory reactions related to JNK and p38 MAPK. In the NP inflammatory microenvironment, the binding of IL-17α to the IL-17 receptor triggers JNK and p38 MAPK activation, thereby inducing the phosphorylation of c-Jun and translocation of c-fos and c-Jun. Subsequently, c-fos and c-Jun synergistically bind AP-1 to promote the expression of cyclooxygenase (Cox)2 and prostaglandin E (PGE)2, thereby increasing inflammatory responses [84]. In contrast, IL-10 effectively reduces p38 MAPK phosphorylation and protects IVD from inflammatory responses [85]. In conclusion, JNK and p38 MAPK play a crucial role in the inflammatory responses associated with IDD, underscoring their significance as potential targets for future IDD treatment strategies.

### 5.3. JNK and p38 MAPK Signaling Pathways Accelerate Cellular Senescence

Cellular senescence, a fundamental mechanism that mediates age-related dysfunction and chronic disease, is characterized by irreversible growth arrest in response to various stress stimuli and is considered to be an important cause of IDD [86]. Cellular senescence is associated with the activation of JNK and p38 MAPK in response to various stressors. Heat shock protein 70 (HSP70) represents a class of molecular chaperones that reduces cell damage under stress by preventing protein denaturation and promoting refolding of denatured proteins. Zhang et al. [20] established an oxidative stress-induced NPC degeneration model and found that HSP70 delayed cellular senescence mediated by p53/p21 by downregulating the JNK/c-Jun pathway. In addition, activation of p38 MAPK by abnormal mechanical stress accelerates senescence in NPCs. In a porcine IVD pressure-intensity culture system, the NPCs were exposed to intermittent dynamic high-intensity compression. The results showed that intermittent high-intensity compression significantly induced the upregulation of senescence-related indices in NPCs in comparison to the control group. However, inhibition of the p38 MAPK pathway reversed the expression of certain senescence markers [21]. Similar results were obtained in rat NPCs, where high-intensity compression forces accelerated the premature senescence of NPCs via the p38 MAPK/ROS pathway [87]. Similar to the mechanical stimuli derived from extracellular sources, intrinsic mechanical signals derived from cells, such as matrix stiffness, are associated with the senescence of NPCs. Zhao et al. [88] cultured rat NPCs in a matrix stiffness model of polyvinyl alcohol hydrogel-simulated normal (4 kPa) and severely degraded (20 kPa) NP tissues with controllable stiffness and the effect of matrix stiffness on senescence was determined by measuring senescence-related indices. The results showed that the expression of p38 MAPK was significantly upregulated in the matrix stiffness model at 20 kPa and promoted the senescence of NPCs. In addition, the levels of lumican derived from the extracellular matrix proteoglycan family increase in degenerated human NP tissues. Lumican blocks the HNPCs cell cycle and accelerates cellular senescence by activating the ASK1/p38 MAPK signaling pathway [89]. Additionally, NPCs were observed to be more senescent under acidic conditions. Fu et al. [90] showed that rat NPCs exhibited a significant decrease in proliferative capacity and telomerase activity at pH 6.2 compared to an acidic environment with pH 7.2, respectively. Additionally, p16 and p53 were upregulated. However, senescence was partially delayed when p38 MAPK activity was inhibited. Therefore, the JNK and p38 MAPK signaling pathways may potentially play key roles in the senescence of NPCs.

### 5.4. JNK and p38 MAPK Signaling Pathways Promote Apoptosis

Apoptosis represents a cell-autonomous and orderly form of cell death regulated by genes to preserve the stability of the intracellular environment; however, abnormal activation of apoptosis can be detrimental to cellular health. In IDD, several apoptotic signals from outside and inside the cell, including extrinsic death receptor, endogenous mitochondrial, and endoplasmic reticulum stress (ERS) pathways, induce IVD cell death [91]. TNF-α is a typical proinflammatory cytokine that activates an extrinsic death receptor pathway. The binding of TNF-α to its receptor upregulates JNK phosphorylation and increases the expression of the C/EBP homologous protein (CHOP). Additionally, it upregulates Bax protein expression and decreases BCL-2 protein levels, thereby inducing apoptosis [92]. Similarly, in TNF-α and IL-1β-treated NPCs, p38 MAPK promotes the activation of caveolin-1/β-catenin signaling, thereby promoting apoptosis [93]. However, recombinant human tumor necrosis factor-α induced protein 6 (RHTSG-6) inhibits the JNK and p38 MAPK signaling pathways and inhibits apoptosis and ECM degradation in HNPCs induced by IL-1β [22]. In addition, mechanical overload induces NPC apoptosis and activates p38 MAPK. However, studies have shown that mechanical growth factor (MGF) protects against mechanical overload-induced apoptosis of NPCs, which is related to the downregulation of the p38 MAPK signaling pathway by MGF [94]. The intracellular ubiquitination level, mediated by p38 MAPK, affects the apoptosis of NPCs. Ubiquitin protein ligase E3 component N-recognin (UBR3) is an E3 ubiquitin-protein ligase involved in the regulation of ubiquitination and studies have shown that UBR3 promotes the ubiquitination degradation of dual specificity phosphatase 1 (DUSP1), which relieves the restriction of p38 MAPK phosphorylation by DUSP1, thus promoting the apoptosis of NPCs [95]. Bacterial IVD injuries are receiving increasing attention from researchers worldwide. *Propionibacterium* acnes has been detected in IVD tissues without suppurative degeneration. P. acnes accelerates the apoptosis of NPCs by activating the Toll-like receptor protein 2(TLR2)/JNK signaling pathway to upregulate the expression of pro-apoptotic proteins [96]. In addition to NPCs, JNK, and p38 MAPK exert pro-apoptotic effects on AF cells [23]. Taken together, these results suggest that inhibiting the activation of the JNK and p38 MAPK signaling pathways may be an effective strategy for mitigating or reversing IVD cell apoptosis.

### 5.5. JNK and p38 MAPK Signaling Pathways Aggravate Oxidative Stress Injury and Lead to Ferroptosis

ROS are byproducts of normal cellular aerobic metabolism and include superoxide anions (O^2−^), hydroxyl radicals (OH−), hydrogen peroxide (H_2_O_2_), and ozone (O_3_). They play diverse roles in various cellular physiological regulation processes. However, excessive ROS accumulation can impair cell function and cause disease. ROS-mediated oxidative stress injury is considered to be an important factor in promoting IDD progression. In NPCs, ROS activates JNK and p38 MAPK pathways. By mediating the activation of these pathways, ROS aggravates the oxidative stress-induced senescence of NPCs, proliferation inhibition, and ECM [24]. In addition, another study revealed that p38 MAPK aggravates the oxidative stress damage process in NPCs under the influence of ROS. Lysine methyltransferase 2D (KMT2D) is a histone methyltransferase essential for fat and muscle cell production, as well as macrophage activation. Studies have shown that p38 MAPK blocks its ubiquitination and degradation by mediating KMT2D phosphorylation under ROS and increasing the expression levels of KMT2D. This leads to the promotion of the expression of catabolic indicators (MMP-3, MMP-9, and MMP-13) and exacerbation of oxidative stress-induced IDD [97]. Quinazoline (QNZ) can mitigate oxidative stress injury in HNPCs by inhibiting the activation of the JNK and p38 MAPK signaling pathways [98]. Furthermore, similar to NPCs, CEP cells undergo significant apoptosis and calcification under oxidative stress, which is associated with the activation of the p38 MAPK signaling pathway [99].

Ferroptosis is a novel, iron-dependent programmed cell death pathway associated with oxidative stress [100]. At its core, the essence of this process is cell death caused by the excessive accumulation of iron-dependent lipid peroxidation, which is one of the pathogenic factors in numerous degenerative diseases. Ferroportin (FPN) is a crucial transmembrane protein involved in iron homeostasis. When FPN is lost, intracellular iron accumulation results in ferroptosis. The p38 MAPK may serve as a key regulator of FPN during ferroptosis in NPCs. Lu et al. [25] showed that in a model of oxidative stress-induced degeneration of HNPCs, FPN expression decreased and was accompanied by an increase in intracellular iron levels. Further mechanistic studies revealed that the activation of p38 MAPK induced by tert-butyl hydroperoxide (TBHP) reduced the nuclear translocation of metal-regulated transcription factor 1(MTF1), resulting in the arrest of FPN transcription. Therefore, inhibition of the p38 MAPK signaling pathway may be a practical therapeutic approach for inhibiting ferroptosis in IVD cells.

### 5.6. JNK and p38 MAPK Signaling Pathways Regulate Autophagy

Autophagy is an essential process in cell metabolism that plays a vital role in cell differentiation, development, survival, and homeostasis. Autophagosomes degrade molecular and subcellular components, including nucleic acids, proteins, lipids, and organelles, by binding to lysosomes to form autophagolysosomes [101]. Autophagy is a double-edged process. In general, upregulation of autophagy favors IVD cell survival; however, excessive autophagy may also aggravate cell damage [64,101].

JNK and p38 MAPK regulate autophagy and affect the biological activity of IVD cells. Under inflammatory conditions, inhibition of JNK activation activates autophagy to alleviate the catabolic processes of the ECM in rat NPCs [102]. Similarly, in a tension load (TL)-induced model of rabbit cartilage endplate stem cell (CESC) degeneration, the JNK signaling pathway was activated and accompanied by a decrease in autophagy levels. When JNK was inhibited, the protein levels of the autophagy markers microtubule-associated protein 1 light chain 3 beta-II (LC3-II) and Beclin-1 increased, while the protein levels of p62 decreased. This suggests that the downregulation of JNK may mitigate TL-induced CEP degeneration through the activation of autophagy [26]. Additionally, p38 MAPK is considered to play a crucial role in inhibiting the activation of autophagy. In a model of oxidative stress-mediated degeneration of NPCs, the p38 MAPK inhibitor, SB203580, relieved the restriction of p38 MAPK on autophagy activation, protected NPCs from apoptosis, and restored ECM homeostasis [27].

Contrastingly, JNK activates autophagy. A study demonstrated that under stress conditions, the phosphorylation of p38 MAPK in rat NPCs was increased, accompanied by the activation of autophagy. The inhibition of JNK signaling leads to impaired autophagy-mediated removal of damaged mitochondria, which in turn increases ROS production and exacerbates compression stress-induced cell damage. This suggests that activation of autophagy by JNK positively influences the compression stress-induced death of NPCs, which may serve as a protective response to stress stimuli [103]. We hypothesized that the dual effects of JNK on autophagy regulation may be related to the different degrees of JNK stress stimulation in cells and the activation of different subtypes of JNK. In conclusion, a comprehensive analysis of the role of JNK and p38 MAPK in IDD through the regulation of autophagy requires further investigation.

## 6. Regulation of p38 MAPK by Non-Coding RNAs in IDD

Noncoding RNAs, mainly microRNAs (miRNAs), circular RNAs (circRNAs), and long noncoding RNAs (lncRNAs), participate in various cellular activities by binding to DNA, RNA, and proteins. Since non-coding RNAs are involved in complex regulatory roles in degenerated IVD cells and crosstalk among various non-coding RNAs has been extensively studied, it has been suggested that they may be utilized as reliable biotherapeutic agents for the development of effective treatment strategies for IDD [104]. It has been observed that various ncRNAs exert regulatory effects on p38 MAPK in IDD.

The miRNAs are small RNAs, approximately 20–24 nucleotides in length, that are widely studied and play multiple important regulatory roles in cells. Li et al. have reported a negative correlation between miRNA-148a and p38 MAPK. It has been reported that p38 MAPK is inhibited when miRNA-148a is overexpressed and is accompanied by a decrease in proinflammatory cytokine levels (TNF-α, IL-1β, and IL-6) [105]. In contrast, miRNA-27a expression exhibits a positive correlation with p38 MAPK expression. The inhibition of miRNA-27a has been reported to inhibit the release of proinflammatory cytokines in degenerated HNPCs by modulating the p38 MAPK signaling pathway [106].

The lncRNAs are a class of non-coding RNAs, which are longer than 200 nucleotides in length and play crucial roles in various cellular processes. Metastasis-associated lung adenocarcinoma transcript-1(MALAT1) is a highly conserved lncRNA that is closely associated with the initiation and development of bone and cartilage diseases [107]. Diabetes mellitus (DM) is a pathogenic condition associated with IDD. The expression of MALAT1 and p38 MAPK was upregulated in a high glucose-induced degeneration model of CEP in rats and was accompanied by different degrees of apoptosis. After the knockdown of the MALAT1 gene, the expression of both total p38 MAPK and phosphorylated p38 MAPK was inhibited and the extent of apoptosis in CEP cells was significantly ameliorated [108]. This finding suggests that MALAT1, a critical regulatory gene of p38 MAPK, holds potential therapeutic significance in managing DM-induced IDD.

In conclusion, targeting p38 MAPK through non-coding RNAs holds potential for the treatment of IDD; however, the present results are preliminary and require further investigation. It is necessary to study the interaction between other non-coding RNAs and p38 MAPK, as well as the crosstalk between non-coding RNAs. In addition, non-coding RNAs that regulate JNK have not been reported for the treatment of IDD and may be worth exploring.

## 7. Potential Therapeutic Strategies Targeting JNK and p38 MAPK Signaling Pathways

**Figure 4:**
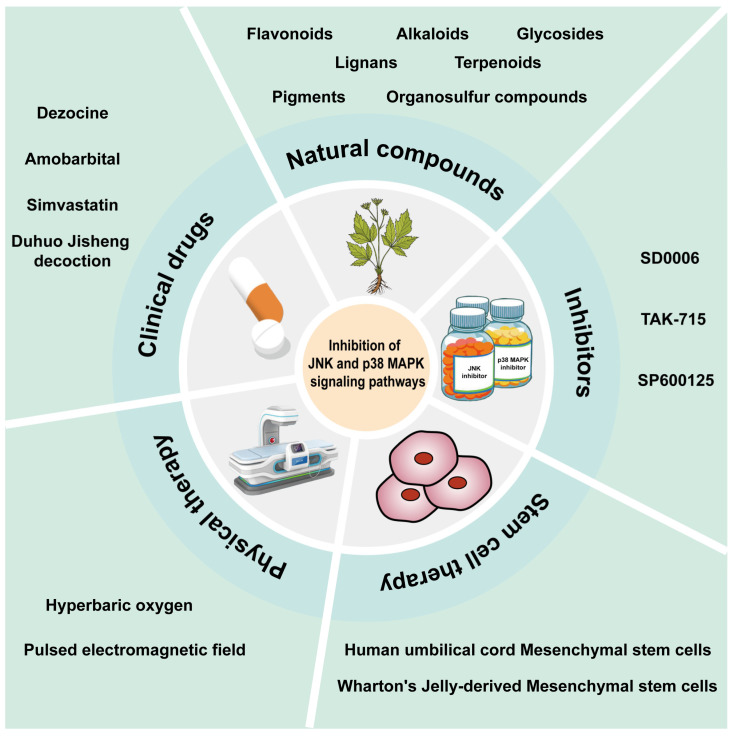
Several therapeutic strategies target the JNK and p38 MAPK signaling pathways. Several common clinical drugs, natural compounds, inhibitors, stem cell therapy, and physical therapy play therapeutic roles in intervertebral disc degeneration by modulating JNK and p38 MAPK.

Currently, the therapeutic strategies targeting JNK and p38 MAPK in IDD are dominated by clinical drugs, natural compounds, inhibitors, stem cell therapy, and physical therapy (Figure 4).

### 7.1. Clinical Drugs

Several drugs commonly used in clinical practices hold potential therapeutic prospects for alleviating IDD through the modulation of JNK and p38 MAPK signaling pathways (Table 1).

Dezocine is a newly synthesized opioid kappa receptor partial agonist with a structure similar to that of pentazocine and is commonly used in perioperative pain management. Dezocine has been shown to delay complete Freund’s adjuvant-induced inflammatory pain in rats and is associated with MAPK family kinase activity. To investigate whether dezocine could modulate inflammation-induced IDD, Zhu et al. [109] established a model of pro-inflammatory cytokine-induced degeneration in HNPCs and administered different concentrations of dezocine. The results showed that dezocine downregulated the expression levels of IL-6, TNF-α, ROS, cleaved-caspase-3, cleaved-caspase-9, and Bcl-2-associated X protein (Bax) in a dose-dependent manner and upregulated superoxide dismutase (SOD)1 and SOD2. Simultaneously, the phosphorylation of p38 MAPK was inhibited. However, the protective effect of dezocine on the IVD was weakened by the addition of the MAPK agonist Phorbol 12-myristate 13-acetate (PMA).

Similar to phenobarbital, amobarbital is a medium-acting hypnotic drug primarily used for hypnosis, sedation, anticonvulsants, and pre-anesthetic administration. As a significant source of ROS, the mitochondrial electron transport complex I can be reversibly inhibited by amobarbital. However, whether amobarbital can improve the level of oxidative stress in IDD and participate in related regulatory mechanisms remains unclear. To address this, Seol et al. [110] established a TBHP-induced IDD model to explore the role of amobarbital. The number of apoptotic and necrotic cells increased in HNPCs treated with TBHP. Conversely, in this model, the number of injured cells was significantly reduced and the mitochondrial membrane potential was significantly enhanced by the addition of amobarbital. Furthermore, amobarbital inhibited TBHP-induced activation of JNK and p38 MAPK, which was not significantly different from the results in the group treated with JNK and p38 MAPK inhibitors. Therefore, amobarbital can alleviate ROS-induced IDD by inhibiting JNK and p38 MAPK. Simvastatin is a lipid-lowering statin that controls blood cholesterol levels and prevents cardiovascular disease. Recent studies have shown that simvastatin can delay the progression of osteoarthritis (OA) and IDD through non-lipid-modulating pharmacological effects [128,129]. Tu et al. observed that simvastatin at concentrations of 10 and 20 μmol/L significantly reduced the protein expression levels of ECM catabolic markers (MMP-3, MMP-13, ADAMTS-4, and ADAMTS-5) and upregulated the expression levels of anabolic markers (type II collagen and aggrecan) in HNPCs under IL-1β induction. Simultaneously, the level of apoptosis significantly improved after simvastatin treatment (flow cytometry showed a 14.59% reduction in the rates of early and late apoptosis). Similarly, it has been reported that JNK and p38 MAPK phosphorylation is limited by simvastatin [111].

Duhuo Jisheng decoction (DHJSD), a traditional Chinese medicine, is frequently employed to effectively treat lower back pain with positive therapeutic outcomes. Liu et al. [112] constructed a stress-induced IDD model and demonstrated that DHJSD promoted NPC proliferation in a time- and dose-dependent manner under static pressure and inhibited ECM destruction and apoptosis. To further explore whether p38 MAPK signaling was involved in the protective effect of DHJSD on NPCs, the HNPCs were pretreated with anisomycin, a p38 MAPK activator. The results showed that anisomycin upregulated the phosphorylation of p38 MAPK and abolished the rescue of ECM metabolic imbalance and the inhibition of apoptotic gene synthesis by DHJSD.

In summary, drug therapy for IDD has tremendous clinical significance. Notably, inhibition of the JNK and p38 MAPK signaling pathways may be a potential target for the clinical pharmacotherapy of IDD.

### 7.2. Natural Compounds

Natural compounds are unique active compounds isolated from plants and animals. Owing to their unique advantages, such as wide distribution, easy-to-obtain materials, and diverse structures, they have excellent potential for the treatment of IDD [130]. To date, natural compounds associated with JNK and p38 MAPK in IDD research are mainly flavonoids, alkaloids, lignans, pigments, terpenoids, glycosides, and organosulfur compounds (Table 1).

#### 7.2.1. Flavonoids

Engeletin is a flavonoid glycoside extracted from the leaves of Engelhardia roxburghiana Wall. It has been widely studied for the treatment of inflammatory diseases. In a rat model of NP degeneration, engeletin delayed IDD progression by downregulating the phosphorylation of JNK and p38 MAPK and inhibiting the generation of inflammatory mediators, apoptotic proteins, and ECM catabolites [113]. Similarly, epigallocatechin 3-gallate (EGCG), an active component of tea polyphenols, inhibits inflammatory responses within NPCs and alleviates nerve root pain in rats, which may be related to the blocked activity of JNK and p38 MAPK [114]. Genistein is an isoflavone extracted from legumes and is often used in antitumor research. The inhibition of p38 MAPK by genistein has been demonstrated in many diseases but its effect is unknown in IDD. Ge et al. [115] observed that genistein delayed the inflammatory response by interfering with NPCs in vitro, which was associated with the inhibition of p38 MAPK phosphorylation. In addition, in vivo studies revealed that after four weeks of genistein treatment, MRI and histological changes were not significant in the low-dose group but were significant in the high-dose group and were accompanied by decreased expression of p38 MAPK. Quercetin is a flavonol that is widely distributed in plants and has anti-oxidative, anti-inflammatory, and anti-apoptotic effects. In a TBHP-treated NPCs degeneration model, quercetin alleviated IDD by inhibiting autophagy activation via the p38 MAPK signaling pathway [27]. Similarly, acacetin, a flavonoid mainly used for antioxidant, anti-inflammatory, and antibacterial purposes, improved TBHP-induced production of inflammatory mediators (Cox-2 and inducible nitric oxide synthase (iNOS)), degradation of the ECM, and upregulation of the expression of antioxidant proteins (heme oxygenase (HO)-1, NAD(P)H: quinone oxidoreductase (NQO)1, and SOD). Acacetin partially delayed IDD by inhibiting JNK and p38 MAPK phosphorylation [116]. Therefore, flavonoids have the advantage of affecting JNK and p38 MAPK signaling in IDD. Additionally, further exploration of other flavonoids and their joint action may potentially be a promising new strategy for the treatment of IDD.

#### 7.2.2. Alkaloids

Berberine and piperine are natural alkaloid compounds that have similar biological effects such as anti-oxidation, anti-inflammation, and autophagy regulation. Berberine is a quaternary ammonium alkaloid that is isolated from Coptis chinensis. Luo et al. [117] showed that berberine inhibited the activation of ERS and autophagy in HNPCs induced by H_2_O_2_, ameliorating oxidative stress-induced apoptosis. Further studies showed that this protective mechanism involved the inhibition of ERS-mediated autophagy activation and inhibition of JNK activity. Thus, JNK may play an important role in this regulatory process. Inositol-requiring enzyme 1 (IRE1), which is expressed on the endoplasmic reticulum membrane when the ER is stimulated by stress, is activated and the activated IRE1 phosphorylates downstream JNK to activate autophagy. However, the activation of ERS-induced autophagy was partially reversed by the JNK inhibitor, SP600125, similar to the effect of berberine. Therefore, berberine may block autophagy activation by ERS and alleviate IDD by inhibiting the JNK signaling pathway. Piperine is an alkaloid derived from pepper. A previous study showed that piperine could inhibit LPS-induced JNK phosphorylation, thus partially alleviating the inflammatory response and ECM imbalance within NPCs. Notably, piperine exhibited no significant effect on LPS-induced phosphorylation of p38 MAPK, presumably because of differences in cell types [118]. In conclusion, the effects of alkaloid compounds on the activities of JNK and p38 MAPK in IDD, especially the mechanism of action of p38 MAPK, require further investigation.

#### 7.2.3. Lignans

Honokiol is a bioactive lignan compound whose primary functions include antioxidant, anti-inflammatory, anti-angiogenic, and antitumor effects. In a study on how honokiol protects against IDD, Tang et al. found that honokiol inhibited JNK phosphorylation more significantly over time. Inhibition of the JNK signaling pathway attenuated the inflammatory response to NPCs [119]. Sesamin is an anti-inflammatory and antioxidant lignan. Sesamin inhibited LPS-induced NPCs proinflammatory cytokines, chemokines, and MΦ migration in a rat model of NPCs degeneration, which may be related to the inhibition of JNK phosphorylation [120].

#### 7.2.4. Pigments

Pigments are a varied group of chemical substances found in biological cells, exhibiting numerous types and sources comprehensively. They are characterized by their high safety, nutritional value, and pharmacological functions. Common natural pigments found in plants and microorganisms include anthocyanins, carotenoids, lycopene, chlorophyll, and monascus. Crocin is a water-soluble carotenoid derived from Crocus sativus that has anti-inflammatory effects on cartilage. Studies have shown that crocin has anti-inflammatory and anti-ECM catabolic effects on NPCs; these mechanisms of action are associated with the impaired activation of JNK by crocin, suggesting that inhibition of the JNK signaling pathway may be a key regulator of crocin-delayed IDD [121]. In addition, other pigment compounds have been reported in the mechanistic studies of IDD; however, the role of these pigments in the context of JNK and p38 MAPK signaling pathways has not been studied extensively.

#### 7.2.5. Terpenoids

Tanshinone IIA is a terpenoid derived from the rhizomes of Salvia miltiorrhiza, a labial plant. It is used to treat cardiovascular diseases and has anti-inflammatory, vasodilating, antihypertensive, and antithrombotic properties. A previous study reported that tanshinone IIA inhibits JNK and p38 MAPK activation induced by IL-1β and affects signal transduction pathways. In particular, the inhibition of p38 MAPK phosphorylation significantly affects the expression of proinflammatory mediators and MMPs, alleviating the inflammatory response in IDD. The anti-inflammatory properties of tanshinone IIA make it an effective pain reliever, capable of alleviating radicular pain induced by IDD. The team tested the pain threshold of the nerve root induced by mechanical stimulation in rats using the von Frey filament test. The results demonstrated that tanshinone IIA significantly increased the threshold for mechanical withdrawal, with the threshold primarily reaching the same level as that observed in the sham-operated group [122].

Notably, tanshinone IIA was found to exert a more potent inhibitory effect on p38 MAPK activity. In a recent study, Dai et al. [123] found that the p38 MAPK pathway was inhibited in the tanshinone IIA-treated group in a degenerated animal model, which was accompanied by the upregulation of antioxidant enzyme activity and downregulation of pro-inflammatory cytokines. These results suggest that targeting the p38 MAPK signaling pathway through tanshinone IIA may hold potential as a therapeutic approach for treating IDD.

#### 7.2.6. Glycosides and Organosulfur Compounds

Glycyrrhizin is an anti-inflammatory and antiviral glycoside that is mainly found in the rhizomes of Glycyrrhiza uralensis Fisch. Liu et al. [124] demonstrated that in a model of pro-inflammatory cytokine-induced degeneration of HNPCs, glycyrrhizin delayed IDD initiation and development by interfering with the JNK and p38 MAPK signaling pathways. This led to the inhibition of high-mobility group box 1 (HMGB1)-induced inflammation and apoptosis within HNPCs. Allicin, a common organosulfur compound, is an active ingredient in garlic exerting anti-senescence, antibacterial, and antioxidant effects. In a study of IDD mediated by oxidative stress, allicin was found to exhibit a significant inhibitory effect on p38 MAPK phosphorylation. In addition, it alleviated the impaired proliferation and mitochondrial apoptosis of HNPCs stimulated by oxidation products in a time- and dose-dependent manner. However, this protective effect was reversed when the cells were treated with a p38 MAPK agonist [125]. Therefore, allicin may alleviate oxidative stress-induced IDD via the p38 MAPK signaling pathway.

### 7.3. Inhibitors

SD0006 and TAK-715 are two common potent inhibitors of p38 MAPK activity, whereas SP600125 is a novel selective inhibitor of JNK (Table 1). Early studies focused on the efficacy of p38 MAPK inhibitors in IDD-induced radicular pain [131,132]. However, with the development of IVD pathophysiology, researchers have focused their attention on the underlying mechanisms within the NPCs. A study conducted earlier reported that SD0006 promotes NPCs proliferation and directly reduces the inflammatory response by activating p38 MAPK [126]. Similarly, a recent study suggested that TAK-715 might be a potential inhibitor that could be used for the treatment of IDD since TAK-715 blocked IL-1β-induced production of inflammatory mediators and inhibited ECM degradation. In vivo, the injection of TAK-715 significantly improved puncture-induced IDD compared to the surgery group according to MRI and histological analysis at eight weeks [127]. In addition, the JNK inhibitor SP600125 is essential for promoting IVD cell proliferation, inhibiting pro-inflammatory cytokine expression, and maintaining ECM stability [19,133]. However, SP600125 can also inhibit autophagy-mediated removal of damaged mitochondria by inhibiting the JNK pathway, thereby increasing ROS production and exacerbating compressive stress-induced NPC damage [103]. This may be related to the conflicting roles of autophagy at different stages of degeneration. In conclusion, JNK and p38 MAPK inhibitors represent promising therapeutic agents for developing effective treatment strategies for IDD. However, further development and screening of effective inhibitors exhibiting definite efficacy and optimization of their safety profiles are required.

### 7.4. Stem Cell Therapy

In recent years, stem cell therapy has experienced rapid and significant advancements in the field of medicine. It is a regenerative medicine system that is designed to repair damaged cells by reducing inflammation and regulating the immune system. There are two main types of stem cells: embryonic stem cells (ESCs) and adult stem cells (ASCs). Mesenchymal stem cells (MSCs) are ASCs that differentiate into bone, cartilage, and fat cells and have been used to treat autoimmune diseases, degenerative bone and joint diseases, and neurological disorders. To investigate the impact of MSCs on high glucose-induced nucleus pulposus mesenchymal stem cells (NPMSCs), Qi et al. cultured NPMSCs by deriving them from human umbilical cord mesenchymal stem cells (hUCMSC). The collected supernatant medium, referred to as mesenchymal stem cell-conditioned medium (MSC-CM), was subsequently used to culture NPMSCs. The results showed that MSC-CM reduced the apoptotic rate of NPMSCs induced by high glucose and rescued ECM degradation, which was related to the inhibition of p38 MAPK phosphorylation by MSC-CM [134].

Similarly, Wharton’s jelly-derived MSCs (WJ-MSCS) were isolated from the umbilical cords and co-cultured with NPCs to explore the protective effects of IVD in an inflammatory setting. Several studies have reported that WJ-MSCs significantly inhibit TNF-α-induced phosphorylation and pro-inflammatory cytokines of p38 MAPK and increase ECM synthesis protein expression, mitigating inflammatory disruption within IDD [135]. In addition, stem cell therapies targeting JNK in IDD have not yet been reported, which may be a direction that researchers should consider and explore.

### 7.5. Physical Therapy

Physical therapy involves the use of electricity, light, magnetism, and heat from natural or artificial sources to prevent or treat diseases. Hyperbaric oxygen therapy (HBO) is a typical physical therapy used to improve tissue and microvascular oxygen levels. Several studies have shown that HBO ameliorates the inflammatory response, deranged ECM metabolic balance, and mitochondrial apoptosis in degenerative NPCs by inhibiting the phosphorylation of JNK and p38 MAPK [136,137,138]. Pulsed electromagnetic field (PEMF) therapy is a physical modality that involves delivering low-frequency electromagnetic waves to target tissues, effectively promoting healing, and providing pain relief. This treatment has demonstrated favorable clinical efficacy for OA [139]. To date, there are no clinical reports on the use of PEMF in the treatment of IDD. Relevant studies have shown that PEMF can reduce the expression of inflammation-related genes involved in IDD; however, the intrinsic pathway remains unclear [140]. Tang et al. [141] observed that the phosphorylation levels of p38 MAPK were significantly inhibited after four hours of treatment with NPCs in an inflammatory microenvironment with PEMF. Further studies have shown that PEMF inhibited the expression of IL-6 through the p38 MAPK pathway, thus controlling the inflammatory response in NPCs. In the future, these non-invasive, low-cost, effective, and safe physiotherapy approaches targeting JNK and p38 MAPK hold the potential to pave the way for new directions in preventing and treating IDD.

## 8. Conclusions and Future Directions

The occurrence and development of IDD involves a series of stress response processes linked to the activation of SAPKs. JNK and p38 MAPK, integral members of the SAPKs family, serve as critical intracellular relay stations in response to various extracellular stress stimuli, consequently transmitting further stress signals to the nucleus or cytoplasm. In degenerated IVD cells, JNK and p38 MAPK signaling pathways are significantly activated and are involved in ECM metabolic balance, inflammatory response, apoptosis, senescence, oxidative stress damage, iron death, and autophagy regulation. This suggests that the activation of JNK and p38 MAPK could potentially be a critical step in IDD. However, the specific mechanisms of action of JNK and p38 MAPK in IDD and the conflicting regulatory roles that arise under different conditions require further investigation. Furthermore, careful consideration should be given to whether the distinct subtypes of JNK and p38 MAPK demonstrate consistent behavior in different IDD cells or if their functions within the same cells may vary.

Currently, common clinical drugs, natural compounds, inhibitors, stem cell therapy, and physical therapy targeting JNK and p38 MAPK effectively protect IDD. However, there are several important considerations. (1) Strategies to minimize long-term side effects of clinical drugs and therapeutic doses. (2) The potential limitations in vivo regarding the activity, specificity, and pharmacokinetic properties of natural compounds. (3) The necessity for extensive preclinical trials to ensure the safety of JNK and p38 MAPK inhibitors in clinical applications. (4) The potential negative impact of stem cell therapy-induced tumorigenesis on degenerated IVD cells. In addition, the regulation of JNK and p38 MAPK activities through tissue engineering and epigenetics may be potentially explored in the future for the development of effective treatment strategies against IDD. In conclusion, JNK and p38 MAPK are potential targets for IDD therapy.

## Figures and Tables

**Table 1:** Potential clinical drugs, natural compounds, and inhibitors targeting JNK and p38 MAPK in intervertebral disc degeneration.

Classification	Name	Structural Formula	Dose/Concentration	Model Construction	Target/ Pathway	Function	References
Clinical drugs	Dezocine	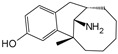	0.5 μg/mL, 1 μg/mL, 2 μg/mL	IL-1β (10 ng/mL) stimulates HNPCs for 24 h	p38 MAPK and ERK1/2	Protect against IL-1β-induced inflammation, oxidative stress, and apoptosis of HNPCs	[109]
	Amobarbital	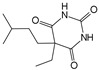	2.5 mmol/L	TBHP (50 μmol/L) stimulates rabbit NPCs for 1 h	ERK, JNK and p38 MAPK	Improved mitochondrial function and decreased TBHP-induced apoptosis, necrosis, and ROS production	[110]
	Simvastatin	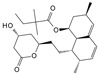	10 μmol/L, 20 μmol/L	IL-1β (10 ng/mL) stimulates HNPCs for 48 h	ERK, JNK and p38 MAPK	Inhibition of IL-1β-induced apoptosis and ECM degradation	[111]
	Duhuo Jisheng decoction		200 μg/mL	HNPCs were subjected to 1 MPa static pressure	p38 MAPK	Attenuate pressure-induced apoptosis and increase ECM synthesis	[112]
Natural compounds	Engeletin	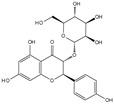	80 μmol/L	TNF-α (20 ng/mL) stimulates rat NPCs for 24 h	ERK, JNK, p38 MAPK and NF-κB	Inhibition of TNF-α-induced apoptosis, inflammation, and ECM degradation	[113]
	Epigallocatechin 3-gallate	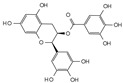	10 μmol/L	IL-1β (10 ng/mL) stimulates HNPCs for 2 h	IRAK-1, p38 MAPK, JNK and NF-κB	Repress inflammatory responses and reduce MMPs	[114]
	Genistein	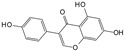	10 ng/mL, 20 ng/mL, 30 ng/mL	IL-1β (10 ng/mL) stimulates rat NPCs for 24 h	P38 MAPK	Relieve IL-1β-induced generation of inflammatory mediators	[115]
	Quercetin	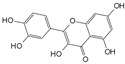	25 μmol/L	TBHP (50 μmol/L) stimulates rat NPCs for 24 h	P38 MAPK, mTOR	Protect against TBHP-induced rat NPC apoptosis and ECM degradation	[27]
	Acacetin	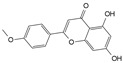	0.3 μmol/L, 1 μmol/L	TBHP (50 μmol/L) stimulates rat NPCs for 24 h	Nrf2, p38 MAPK, JNK and ERK1/2	Relieve TBHP-induced generation of inflammatory mediators and ECM degradation	[116]
	Berberine	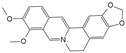	1 μmol/L, 2 μmol/L, 4 μmol/L, 8 μmol/L	H_2_O_2_ (300 μmol/L) stimulates HNPCs for 24 h	IRE1 and JNK	Prevent H_2_O_2_-induced apoptosis by modulating ER stress and autophagy	[117]
	Piperine	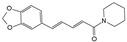	10 μmol/L, 50 μmol/L, 100 μmol/L	LPS (10 μg/mL) stimulates rat NPCs for 24 h	JNK and NF-κB	Protect against LPS-induced inflammation and ECM catabolism	[118]
	Honokiol	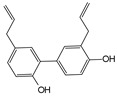	2.5 μmol/L, 5 μmol/L	H_2_O_2_ (500 μmol/L) stimulates rat NPCs for 24 h	TXNIP, NLRP3, JNK and NF-κB	Reduce H_2_O_2_-induced oxidative stress, apoptosis, and inflammatory responses	[119]
	Sesamin	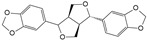	0.1 μmol/L, 0.5 μmol/L, 1 μmol/L	LPS (10 μg/mL) stimulates rat NPCs for 24 h	JNK	Inhibition of LPS-induced inflammatory responses and ECM catabolism	[120]
	Crocin	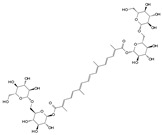	10 μmol/L, 50 μmol/L, 100 μmol/L	LPS (10 μg/mL) stimulates rat NPCs for 0.5 and 24 h	JNK	Repress LPS-induced inflammatory responses and reduce ECM catabolism	[121]
	Tanshinone IIA	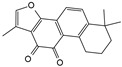	1. 10 or 20 mg/kg body weight; 2. 5 μmol/L	1. Percutaneous needle puncture of rat caudal vertebra; 2. NPCs were derived from human degenerative IVD tissue	1. P38 MAPK 2. IRAK-1, p38 MAPK, JNK and NF-κB	1. Reduce damage—induced oxidative stress and IDD 2. Suppress inflammatory responses and reduce MMPs	[122,123]
	Glycyrrhizin	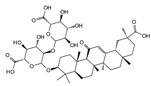	100 μmol/L	IL-1β (10 ng/mL) stimulates HNPCs for 24 h	p38 MAPK, JNK and HMGB1	Inhibition of IL-1β-induced cell apoptosis and inflammatory responses	[124]
	Allicin	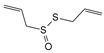	5 μmol/L, 10 μmol/L, 20 μmol/L	AOPP (400 μg/mL) stimulates HNPCs for 24 h	P38 MAPK	Attenuate AOPP-induced oxidative stress and mitochondrial apoptosis	[125]
Inhibitors	SD0006	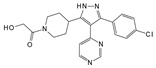	70 nmol/L	IL-1β (5 ng/mL) stimulates HNPCs for 24 h	p38 MAPK	Reduce IL-1β- induced cell proliferation inhibition and inflammatory responses	[126]
	TAK-715	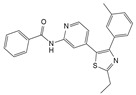	0.5 μmol/L, 1 μmol/L	IL-1β (10 ng/mL) stimulates rat NPCs for 48 h and IL-1β-induced ex vivo IVD culture model	P38 MAPK	Protect against IL-1β-induced rat NPC apoptosis and ECM degradation	[127]
	SP600125	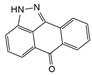	10 μmol/L	HAFCs and HNPCs were co-cultured with phorbol myristate acetate-stimulated macrophage-like THP-1 cells	JNK	Attenuate co-culture cells-induced inflammatory responses	[19]

Abbreviations: NPCs, Nucleus pulposus cells; IVD: Intervertebral disc; IDD, Intervertebral disc degeneration; HNPCs, Human nucleus pulposus cells; HAFCs, Human annulus fibrosus cells; H_2_O_2_, Hydrogen peroxide; TBHP, Tert-butyl hydroperoxide; ROS, Reactive oxygen species; ECM, Extracellular matrix; LPS, Lipopolysaccharide; TNF-α, Tumor necrosis factor-alpha.

## Data Availability

Not applicable.
